# Hiding in plain sight: A call to prevent cutaneous leishmaniasis transmission in the United States

**DOI:** 10.1371/journal.pntd.0014297

**Published:** 2026-05-08

**Authors:** Juan David Ramírez, Sarah M. Gunter, Megan Coffee, Norman Beatty, Dawn M. Wetzel

**Affiliations:** 1 Center for Global Health and Interdisciplinary Research, USF Genomics Program, Department of Global, Environmental and Genomic Health Sciences, College of Public Health, University of South Florida, Tampa, Florida, United States of America; 2 Division of Tropical Medicine, Department of Pediatrics, National School of Tropical Medicine, Baylor College of Medicine and Texas Children’s Hospital, Houston, Texas, United States of America; 3 The William T. Shearer Center for Human Immunobiology, Texas Children’s Hospital, Houston, Texas, United States of America; 4 Division of Infectious Diseases and Immunology, Department of Medicine, New York University, New York, New York, United States of America; 5 Department of Population and Family Health, Mailman School of Public Health, Columbia University, New York, New York, United States of America; 6 International Rescue Committee, New York, New York, United States of America; 7 Division of Infectious Diseases and Global Medicine, Department of Medicine, University of Florida College of Medicine, Gainesville, Florida, United States of America; 8 Emerging Pathogens Institute, University of Florida, Gainesville, Florida, United States of America; 9 Department of Pediatrics, University of Texas Southwestern Medical Center, Dallas, Texas, United States of America; 10 Department of Biochemistry, University of Texas Southwestern Medical Center, Dallas, Texas, United States of America; Pure Earth, UNITED STATES OF AMERICA

## Abstract

Cutaneous leishmaniasis (CL), once considered a travel-associated tropical disease, is increasingly transmitted within the United States, particularly in southern regions. Despite mounting evidence of local transmission, public health recognition and preventive infrastructure remain limited. This Viewpoint highlights the urgent need to shift the U.S. CL response from questioning endemicity to preventing transmission. We review ecological, clinical, and surveillance data demonstrating the presence of competent vectors, animal reservoirs, and autochthonous human cases. Diagnostic delays, underreporting, and insufficient provider training contribute to missed prevention opportunities. Climate change and peri-urban rodent-human contact data further heighten future risk. A coordinated response is essential, including national notifiability, expanded diagnostics, integrated vector and reservoir surveillance, clinical education, and One Health–focused research. Without immediate action, CL risks becoming an entrenched, neglected zoonosis in the United States.

## Why cutaneous leishmaniasis (CL) prevention must be a priority

Cutaneous leishmaniasis (CL), an infection caused by *Leishmania* parasites and transmitted by phlebotomine sand flies, causes disfiguring skin lesions and long-term psychosocial consequences. Once considered strictly a tropical disease affecting travelers or deployed military personnel, CL is now being acquired within the U.S., especially in southern states. In fact, a retrospective study in Texas found 59% of 69 CL cases had no international travel history [1]. Pediatric cases in northern Texas have further confirmed the presence of U.S.-specific strains of *Leishmania mexicana* [2]. A broader One Health scoping review of leishmaniasis in Texas identified not only local human transmission but also the ecological presence of vectors and reservoirs essential to sustain disease cycles [3]. A recent work adds another layer of concern by documenting novel genetic strains of *Leishmania* circulating in the U.S. [4].

Despite clear signs of local transmission, public health recognition and intervention for CL remain limited and reactive [1–3]. However, recognized endemicity demands a new public health orientation. Rather than continuing to debate whether CL is endemic, the U.S. should focus on preventing further establishment and spread. When unrecognized or untreated, CL lesions can become chronic and stigmatizing, resulting in unnecessary patient suffering and healthcare costs. From a systems perspective, ongoing transmission represents a failure of surveillance, diagnostics, and education.

## Vector and reservoir ecology: The transmission engine

The ecological components necessary for CL transmission are present in parts of the United States, including the pathogen, competent reservoirs, and established sand fly populations. Sand fly species such as *Lutzomyia anthophora* and *Lu. diabolica* are well documented in Texas and neighboring states and are known to feed on humans [5,6]. In total, at least seven sand fly species have been reported in the U.S. with varying degrees of suspected or demonstrated vector relevance, including *Lu. shannoni, Lu. anthophora, Lu. diabolica, Lu. vexator, Lu. cruciata, Lu. texana,* and *Lu. sanguinaria* [6]. However, confirmed vector competence, defined as experimental evidence of parasite acquisition, development, and transmission, has been established for only a subset of these species. For others, current evidence rests primarily on ecological plausibility, such as geographic overlap with *Leishmania*-positive hosts, human-biting behavior, and phylogenetic relatedness to known vectors. Many of these sand flies have been detected near sites of confirmed human or animal CL cases, yet comprehensive studies of their infection rates, feeding preferences, and transmission efficiency remain limited. Thus, while the structural components of transmission are present, the strength and efficiency of these transmission systems require further empirical clarification. Distinguishing confirmed vector competence from ecological co-occurrence is essential for accurately assessing transmission risk and prioritizing entomological research.

Rodents, particularly *Neotoma micropus*, are established zoonotic reservoirs for *L. mexicana* in the southern United States [7]. Infected rodents typically exhibit minimal or no clinical signs, enabling silent persistence within enzootic transmission cycles. Rodents demonstrate the highest documented infection prevalence (11%) and are strongly implicated in maintaining the sylvatic *L. mexicana* transmission system involving sand flies and humans in endemic foci [3]. This rodent–sand fly–human cycle represents the primary ecological framework for autochthonous cutaneous leishmaniasis (CL) in the U.S.

In contrast, dogs are well-recognized reservoirs for *L. infantum*, the causative agent of visceral leishmaniasis (VL), but they are not considered major reservoirs for *L. mexicana* or other CL agents in most endemic regions. The frequently cited statistic that dogs account for 95.5% of reported animal cases in the U.S. derives from a scoping review encompassing all *Leishmania* species in Texas [8]. Importantly, this figure is driven predominantly by *L. infantum* infections in Foxhound populations, largely maintained through vertical (mother-to-pup) transmission—a transmission dynamic epidemiologically distinct from the sylvatic *L. mexicana* cycle. Presenting these data without clear distinction risks conflating two separate transmission systems. Within a One Health framework, canine *L. infantum* infections should therefore be interpreted as indicators of broader *Leishmania* circulation within shared ecological landscapes, rather than as direct evidence of CL risk. Explicit differentiation between the sylvatic rodent–sand fly–human transmission cycle of *L. mexicana* and the predominantly domestic, vertically maintained canine cycle of *L. infantum* is essential for accurate risk assessment and targeted surveillance strategies.

Other synanthropic mammals that frequently interact with human environments, such as *Didelphis* spp. (opossums), warrant ecological consideration. However, to date, there are no published reports documenting *Leishmania* infection in *Didelphis* species in the United States. This absence highlights a surveillance gap and represents an important area for future One Health research aimed at clarifying the broader mammalian host range of *L. mexicana* in emerging domestic transmission zones.

**Fig 1:**
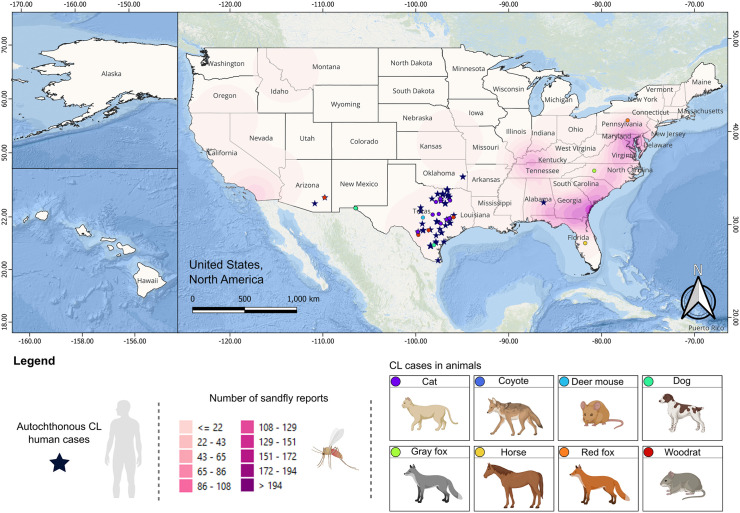
Spatial distribution of autochthonous human and animal cutaneous leishmaniasis cases in the United States, overlaid with sand fly vector report density. Kernel density heatmap (magenta scale) represents the number of reported sand fly occurrences across the United States. Reported cases of cutaneous leishmaniasis are overlaid as point features, with human cases represented by star symbols and animal cases represented by colored circles. The basemap used for geographic visualization corresponds to the ESRI Ocean basemap (*World_Ocean_Base*), available at: https://services.arcgisonline.com/ArcGIS/rest/services/Ocean/World_Ocean_Base/MapServer. This basemap is provided by Esri and is intended for visualization purposes. Licensing and terms of use are available at: https://www.esri.com/en-us/legal/terms/full-master-agreement. United States state boundaries were obtained from the U.S. Census Bureau Cartographic Boundary Files (https://www.census.gov/geographies/mapping-files/time-series/geo/carto-boundary-file.html), which are publicly available and in the public domain, making them compatible with a CC BY 4.0 license. All spatial layers and overlays (sandfly occurrence data and leishmaniasis case reports) were generated and plotted by the authors using geographic information system (GIS) tools in QGIS. Graphical icons used in this figure were created with BioRender.com, Hernandez Valencia, J. C. (2026) https://BioRender.com/stjntlm.

Climate models further support concerns regarding CL’s expansion. Rising temperatures and shifting humidity patterns are predicted to extend the habitat of competent sand fly vectors northward by 2080 [5], which would double the human population at risk. Additionally, changing land use, urban sprawl, and increasing rodent-human interface heighten the potential for human-vector contact (Fig 1).

## Missed diagnoses, missed opportunities

Despite the ecological and epidemiologic evidence of domestic transmission, public health and clinical systems remain insufficiently prepared to address CL. Clinically, CL most often presents as a painless, slowly enlarging ulcer or nodule that closely mimics bacterial, fungal, or neoplastic conditions. Misdiagnosis is therefore common, particularly in patients without a travel history. Importantly, bacterial superinfection of CL lesions is frequent, meaning conventional microbiologic cultures may yield pathogenic bacteria that appear to explain the lesion and justify antibiotic therapy. While appropriate for secondary infection, this can mask the underlying parasitic etiology and contribute to delayed recognition. Studies show that many patients—particularly children—are initially treated with multiple courses of antibiotics, sometimes followed by corticosteroids (which may exacerbate lesions), and only undergo biopsy after months of persistence [2]. In adults, lesions resembling benign dermatologic conditions may be treated empirically with cryotherapy, precluding diagnostic confirmation and obscuring epidemiologic linkage [1]. Conversely, CL lesions may be mistaken for cutaneous malignancy, leading to unnecessarily invasive surgical procedures.

Diagnostic limitations further compound these missed opportunities. Molecular testing (PCR) and culture are largely restricted to specialized reference laboratories in the U.S., and species-level identification—critical for treatment selection—is performed in fewer than one-third of confirmed cases. Even in centers with established molecular capacity, confirmation rates may be incomplete; for example, reported data from the University of Washington School of Medicine indicate that only approximately half of submitted suspected cases were molecularly confirmed [9]. While reference laboratories can return PCR results within several days—comparable to conventional bacterial culture—access remains uneven, and specimen submission pathways are not standardized. The primary barriers are not turnaround time alone, but technological availability, reimbursement structures, and regulatory constraints that limit broader clinical integration. Unlike acute infections requiring immediate “treat-and-release” decisions, CL typically follows a prolonged course, making true point-of-care testing less urgent than reliable access to standardized molecular confirmation. Without consistent diagnostic pathways and species-level identification, cases remain underrecognized and cannot be effectively linked to local transmission networks, limiting public health response and surveillance capacity.

## Gaps in awareness and education

Medical education in the U.S. rarely addresses CL outside global health or travel medicine contexts. As a result, dermatologists, infectious disease specialists, and primary care providers may not routinely consider CL in the differential diagnosis—particularly in patients without recent international travel [1,9]. This diagnostic gap reflects a broader uncertainty: we still lack a clear understanding of risk factors for domestic acquisition. Systematic collection of occupational, environmental, and behavioral exposure data—such as outdoor work, peri-domestic animal contact, land-use proximity, and recreational activities—could substantially strengthen One Health surveillance approaches and inform targeted public health education. Without these data, opportunities to define transmission patterns and identify at-risk populations remain limited.

For migrant communities from Latin America, where CL is endemic, delayed diagnosis may be compounded by structural barriers to healthcare access, limited disease awareness, and stigma [10]. These cases should not be pathologized, but rather recognized as epidemiologically informative and critical for understanding transmission dynamics. In the current sociopolitical climate, concerns about immigration enforcement may further discourage some individuals from seeking timely medical evaluation, creating additional obstacles to early detection and reporting. Broader clinician education that emphasizes inclusive, exposure-based risk assessment—rather than reliance solely on travel history—is essential [14]. Artificial intelligence tools for lesion recognition may eventually support earlier identification; however, these technologies remain underutilized and require rigorous validation before widespread implementation. To date, the only documented digital approach in this context is a smartphone-based application using a validated symptom-based algorithm deployed among community health workers in rural Colombia, rather than image-based artificial intelligence models integrated into routine dermatologic practice [11].

Entomological preparedness also lags behind the evolving epidemiology. Sand flies are not systematically incorporated into national or state-level vector surveillance programs [5,6], creating a critical blind spot in mapping vector distribution and ecological suitability. Integrating occupational risk assessment, clinician awareness, and vector surveillance into a coordinated One Health framework will be necessary to move from reactive case recognition to proactive prevention.

Failure to recognize and respond to U.S. CL transmission carries substantial clinical and public health consequences. Patients may endure chronic, disfiguring lesions, unnecessary surgical procedures, or prolonged pharmacologic treatments before receiving an accurate diagnosis [1,3,9]. At a systems level, missed or delayed diagnoses obscure emerging transmission hotspots, limiting timely public health response, ecological investigation, and targeted resource allocation. Continued framing of CL as exclusively imported or exotic fosters a false sense of security and overlooks risks to domestic populations residing in ecologically suitable regions. Although this discussion centers on CL, many of these concerns extend to other forms of leishmaniasis, particularly visceral leishmaniasis (VL). Unrecognized or latent infections may carry long-term implications, including the potential for recrudescence in individuals who later become immunocompromised. Such risks are especially relevant in the context of solid organ transplantation and other forms of iatrogenic immunosuppression, where undetected infection could result in severe disease. While VL is beyond the primary scope of this manuscript, the broader surveillance, diagnostic, and education gaps described here are not syndrome-specific and may have implications across the leishmaniasis spectrum.

The animal health dimension further underscores the need for a coordinated response. Dogs, horses, and wildlife can be affected, generating emotional and financial burdens for pet owners, veterinarians, and livestock handlers [3,7,15]. A comprehensive One Health framework—integrating human, veterinary, and ecological surveillance—is essential to capture the full burden of disease, identify transmission networks, and prevent further establishment in domestic settings.

## Strategic actions to prevent transmission

To address this growing public health threat, we propose a five-part strategy:

1. **Make CL a Nationally Notifiable Disease.** Mandating CL as a reportable condition nationwide would enable the CDC and local health departments to monitor incidence trends, identify hotspots, and allocate resources appropriately. Texas is currently the only state with mandatory CL reporting, and underreporting remains a major issue [1,3].
2. **Expand Diagnostic Infrastructure.** Strengthening U.S. diagnostic capacity for leishmaniasis should focus on improving access to existing high-quality assays rather than simply expanding the number of testing laboratories. Several high-complexity CLIA laboratories already offer *Leishmania* PCR with strong performance characteristics, and published primer sets are available for broader adoption. The primary barriers are logistical and economic: species-level identification often requires sequencing, maintaining low-volume PCR-plus-sequencing workflows is costly, and many laboratories lack validated positive control materials necessary for CLIA approval. Reliance on centralized federal testing alone is fragile, and public health laboratory pathways may involve complex submission processes and variable turnaround times. Federal support should therefore prioritize validation resources, reference materials, and sustainable funding mechanisms that enable regional high-complexity laboratories to maintain reliable molecular diagnostics. Expanding access to validated existing tools will improve timely diagnosis, species-level identification, and surveillance in emerging U.S. transmission areas. Investment in laboratory infrastructure is essential. The CDC should disseminate PCR protocols to state and county health departments, and support development of rapid diagnostics with higher sensitivity and specificity for U.S. *Leishmania* strains [9,12]. Currently available antigen tests show limited utility for identifying *L. mexicana* or *L. infantum*. Decentralized, species-specific diagnostics will facilitate more accurate treatment and enable epidemiologic surveillance.
3. **Strengthen Entomological, Ecological, and Veterinary Surveillance.** Regular monitoring of sand fly populations, rodent reservoirs, and domestic animals should be implemented in areas with confirmed autochthonous transmission. These data should be incorporated into state-level vector control and climate adaptation plans. Coordinated efforts across public health, veterinary, and wildlife sectors can create a comprehensive map of transmission risk [5–7,13].
4. **Enhance Clinical Education and Public Awareness.** Continuing medical education (CME) modules for dermatologists, infectious disease physicians, and primary care providers should emphasize potential for domestic transmission. Public health campaigns must raise awareness among at-risk communities about preventive measures, such as limiting outdoor exposure during peak sand fly activity periods and reducing peridomestic rodent habitats [10,11,14].
5. **Support Translational and Field Research.** There is an urgent need for research tailored to the U.S. context, including studies on the pathogenic potential of novel *Leishmania* strains, drug resistance profiles, and optimal treatment regimens. Surveillance-based research on animal reservoirs and vector competence is equally critical. Cross-disciplinary collaboration is necessary to develop innovative tools and strategies, from vaccines to integrated vector management [3,7,13,15,16].

## A coordinated path forward

Addressing the emergence of CL in the U.S. demands a coordinated response that integrates human, animal, and environmental health. The first step is recognition of CL as a domestically transmitted disease with potential for further spread. Making CL nationally notifiable, scaling diagnostic access, and expanding clinical education are foundational pillars of any successful response. Vector surveillance and control must be modernized. Sand flies should be included in national and state-level entomological programs, with attention to mapping and climate modeling to predict future risk zones. Surveillance should also extend to wildlife and domestic animals, particularly dogs, which serve as early indicators of human exposure risk.

Although this manuscript focuses on CL, it is important to recognize that several examples cited in discussions of zoonotic spillover and autochthonous transmission—including some northern U.S. cases—are attributable to visceral leishmaniasis (VL). In addition, there is a documented burden of latent or asymptomatic VL among U.S. service members deployed to Iraq and Afghanistan, with reported seroprevalence estimates exceeding 10% in some cohorts. These observations underscore that leishmaniasis in the U.S. extends beyond cutaneous manifestations and reflects a broader, evolving epidemiologic landscape. Importantly, many of the public health solutions proposed for CL—improved diagnostics, standardized disease notification, strengthened veterinary surveillance, and expanded clinical and public education—are equally applicable to VL.

Investment in One Health research will not only improve our understanding of the disease but also drive development of tools to interrupt transmission. Recent studies highlighting novel U.S. strains of *Leishmania* underscore the importance of sustained, context-specific research [2,4]. By uniting efforts across sectors, the U.S. can prevent CL from becoming an entrenched public health issue. Failure to act now risks missing the window to control local transmission. The opportunity exists to leverage existing knowledge, implement targeted interventions, and build a resilient infrastructure capable of addressing CL and other emerging vector-borne diseases. This moment calls for vision, coordination, and decisive action.
